# Pay Up

**Published:** 1883-09

**Authors:** 


### PAY UP.

Many of our subscribers have written, promising to pay their subscriptions in the fall. The fall is now upon us, and we trust they will remember their promises and forward the amount due at once. We have waited patiently during the summer, and now we beg you, gentlemen, to do your part. We cannot publish a journal without money. Let us hear from you at once.

